# Severe major vessel injury during peadicle screw removal: a case report

**DOI:** 10.3389/fsurg.2023.1187801

**Published:** 2023-06-13

**Authors:** ShengYu Fu, Gang Ju, Xiang Dai, Haijun Li, Aibing Huang

**Affiliations:** ^1^ Department of Orthopaedic, The Affiliated Taizhou People’s Hospital of Nanjing Medical University, Taizhou, China; ^2^ Postgraduate School, Dalian Medical University, Dalian, China; ^3^ Department of Vascular Surgery, The Affiliated Taizhou People's Hospital of Nanjing Medical University, Taizhou, China

**Keywords:** pedicle screw, implant removal, iatrogenic vascular injury, inferior vena cava, case report

## Abstract

**Introduction:**

Pedicle screw fixation (PSF) has been the standard therapy for the treatment of various spinal diseases. Although complications are identified regularly, iatrogenic vascular injury is one of the rare but life-threatening complications. In this literature, we describe the first case of inferior vena cava (IVC) injury during pedicle screw removal.

**Case description:**

A 31-year-old man was treated by percutaneous pedicle screw fixation for an L1 compression fracture. After a year, the fracture healed well and hardware removal surgery was performed. During the procedure, the hardware on the right was removed unremarkably except for the L2 pedicle screw which slipped into the retroperitoneum because of the improper technique. The CT angiogram revealed the screw had breached the anterior cortex of the L2 vertebral body and penetrated the IVC. After multidisciplinary cooperation, the defect of IVC was reconstructed and the L2 screw was removed from the posterior approach in the end.

**Result:**

The patient recovered well and was discharged after 3 weeks without further events. The removal of the contralateral implants was unremarkable at 7 months postoperatively. At the 3-year follow-up, the patient returned to his normal daily activity without any complaints.

**Conclusion:**

Although pedicle screw removal is a rather simple procedure, severe complications may have occurred from this procedure. Surgeons should keep vigilant to avoid the complication noted in this case.

## Introduction

The pedicle screw fixation (PSF) has been the standard therapy for the treatment of various spine diseases (1). However, there have been reports on undesired intraoperative and postoperative complications related to PSF (2). Iatrogenic vascular injury is one of the rare but life-threatening complication. Based on the literature, the incidence of large vessel injury caused by pedicle screws is 0.01% (3). Although the majority of pedicle screws related vascular complications occur during the intraoperative period from screw placement, they can also occur in a delayed period from pseudoaneurysm formation (4, 5).

Hardware removal is the most commonly performed orthopedic surgery, the procedure carries risks of unexpected complications (6, 7). According to the reports, the rate of its associated complication was 9.6% in a cohort of recently trained orthopedic surgeons in the United States (8). Although PSF related vessel injuries were well discussed in the literature (9, 10), to our knowledge, there are no reports describing the major vessel injury during pedicle screw removal.

Here, we present a case of inferior vena cava (IVC) injury secondary to the removal of the pedicle screw following percutaneous PSF in treating a thoracolumbar fracture.

## Case description

**Figure 1:**
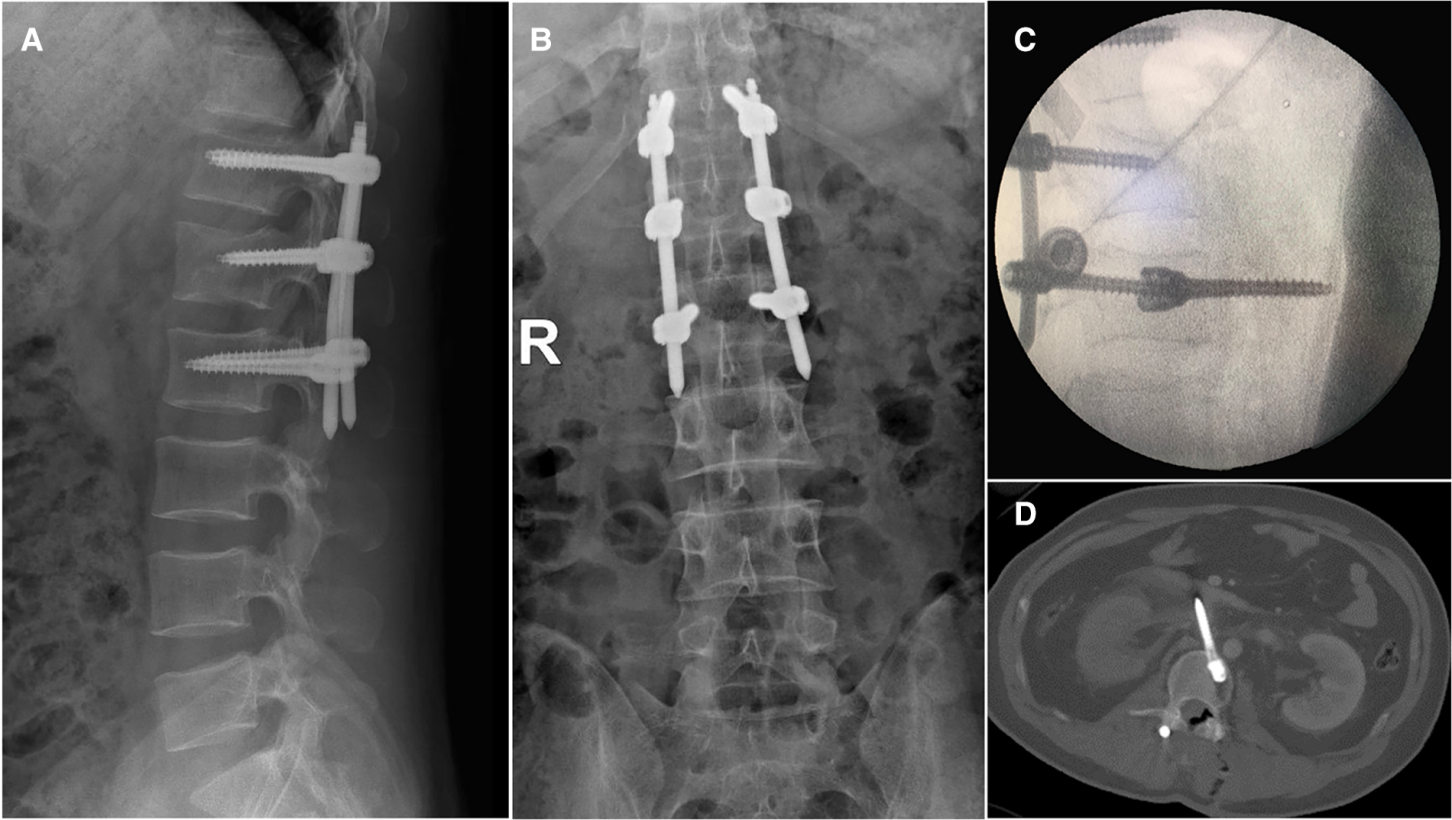
(**A,B**) anterior-posterior and lateral radiographs 1 year after percutaneous pedicle screw in L1. (**C**) Intraoperative fluoroscopy was taken and confirmed that the screw migrated anteriorly to the vertebral body. (**D**) Enhanced CT scanning of the abdomen found that the tip of the right L2 screw indented the IVC and left renal vein without blood extravasation.

A 31-year-old man was treated by percutaneous PSF for an L1 compression fracture. The implant was required to remove one year after the initial surgery without sign of hardware failure or loosening, and bone healing was achieved according to radiography (Figures 1A,B).

Hardware removal surgery was performed in a prone position. During surgery, the caps on the right side was firstly loosened using a remover, and the caps and the rod were removed sequentially, then the pedicle screws of the T12 and L1 were removed unremarkably using the proper screwdriver. However, the removal of the right L2 pedicle screw was unsuccessful. With a limited view of minimal invasive incision, the screwdriver has never been able to fully fit the tail of the screw. After multiple attempts, the screwdriver seemed to fit. To enhance the sense of fit, the surgeon put forwarding pressure on the screw and rotated the screwdriver in a clockwise direction(in a wrong manner). Even worse, he suddenly felt that the screw was pushed into the retroperitoneum.

Intraoperative fluoroscopy was taken and confirmed that the screw migrated anteriorly to the vertebral body (Figure 1C). An endovascular team was emergently consulted. A contrast-enhanced computed tomography (CT) and computed tomography angiogram (CTA) of the abdomen was suggested to assess the extent of vascular injuries. The emergency contrast-enhanced CT and CTA revealed the L2 screw had breached the anterior cortex of the L2 vertebral body and penetrated the IVC but had not caused any extravasation of contrast. (Figure 1D).

**Figure 2:**
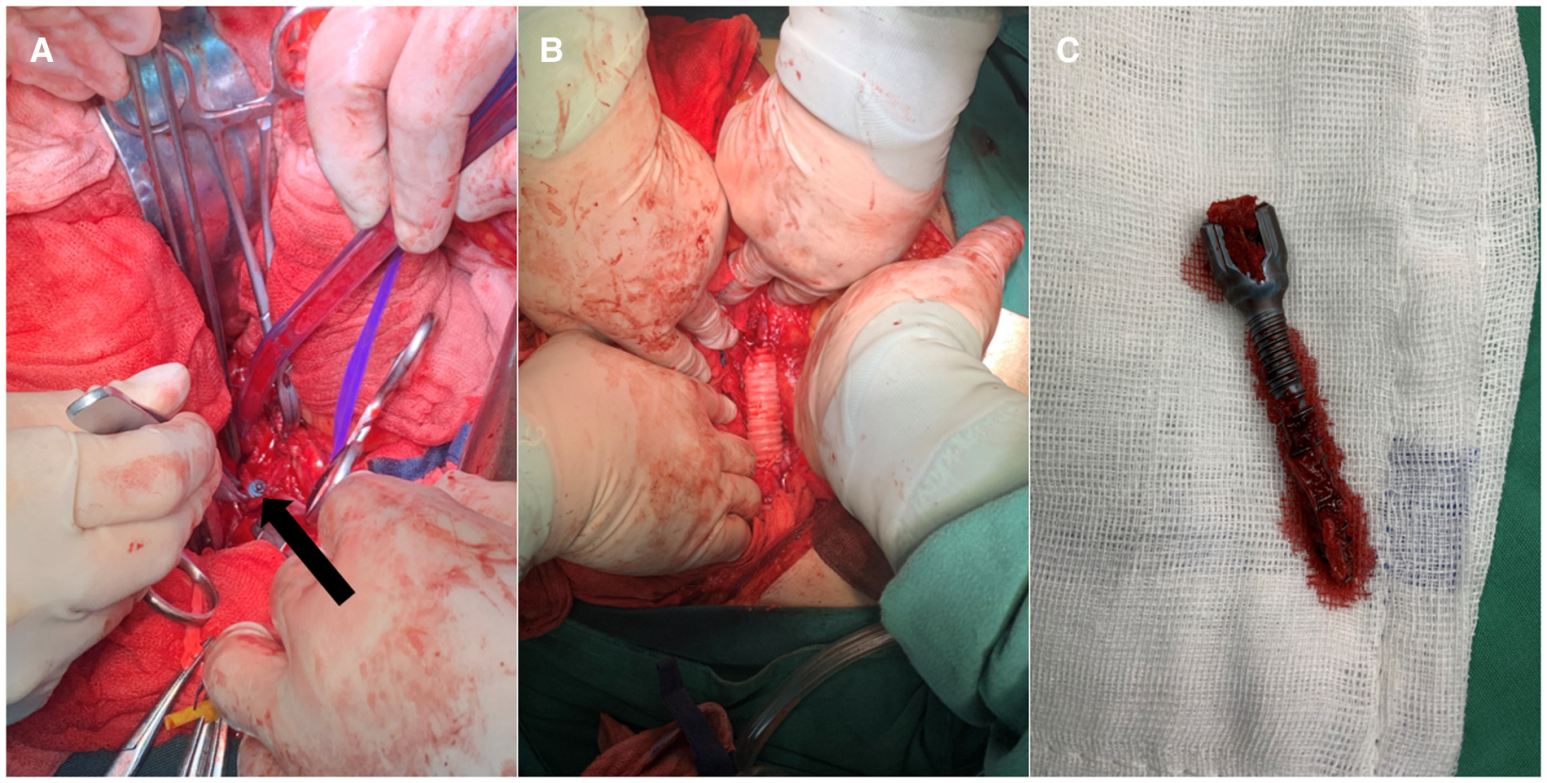
**A** View of the tip of the screw (black arrow) is exposed by the dissection flap of the IVC. (**B**) The defect of IVC was reconstructed using an artificial graft. (**C**) The right L2 pedicle screw was removed in the posterior approach.

The patient was hemodynamically stabilized by fluid resuscitation. Subsequently, a laparotomy was performed in a supine position by a vascular surgeon. The circumference of the IVC wall was evaluated. It was identified that the IVC was traversed, and the bilateral renal veins were also contorted by the screw. After carefully separating the blood vessels from screw, it was found that the IVC was completely punctured and there was a severe crush injury at the transition between the renal vein and the IVC. Vascular control above and below the level of injury was performed, and the screw was slowly backed out (Figure 2A). Finally, the defect of IVC was reconstructed using an artificial graft. (Figure 2B).

Immediately following the abdomen incision was closed, the L2 screw was then removed from the posterior approach (Figure 2C). Considering the general condition of the patient, the removal of the contralateral implants was suspended. At the end of the procedure, he was transferred to the intensive care unit.

He recovered well and was discharged from our institution after 3 weeks without further event. At 7 months postoperatively visit, the removal of the contralateral implants was done with unremarkable. At the 3-year follow-up, the patient returned to his normal daily activity without any complaints. The diagnosis and treatment process of patient were showed in Table 1.

**Table 1:** Patient diagnosis and treatment process.

Time	Clinical situation	Therapeutic method	Supplementary
April 28, 2018	L1 compression fracture	Percutaneous pedicle screw fixation	
April 19, 2019	The fracture healed well	Hardware removal surgery	The IVC was injured by the implant, and then it was repaired.
October 24, 2019	Unremarkable	Removal of the contralateral implant	

## Discussion

PSF has developed into the standard therapy for the treatment of spine diseases (1). After temporary ﬁxation, removal of screws is planned routinely. Although various complications were reported during pedicle screw removal (8), errant IVC injury is a rare but life-threatening complication. To the best of our knowledge, this is the first report of an IVC injury during pedicle screw removal.

Hardware removal remains a controversial topic in spine surgery after a successful fusion (11). Smits et al. reported that quality of life and patient satisfaction improved after implant removal in 108 patients who successfully treated thoracic lumbar fractures (7). Similarly, Chang-Hoon et al. believed that the segmental angular motion of patients can be restored after the removal of internal fixation, which could improve a series of related clinical symptoms (12). Alanay et al. found that after implant removal the rate of functional improvement and VAS decrease was 84%, and 50% respectively (6). However, in a retrospective study, Stavridis et al. found that only 12% of patients experienced complete remission of symptoms after the removal of pedicle screws (13).

Although whether to remove the pedicle screw was controversial, the procedure was still frequently performed. Its associated unexpected complications were occasionally reported. Crasto et al. reported a case of pneumothorax secondary to the broken bolt cutter jaw entering the pulmonary cavity during removal of hardware performed on the thoracolumbar spine, which resulted in the patient death (14). Vanichkachorn et al. described a case that underwent a potential for aorta injury during unsuccessful broken pedicle screw removal in the thoracolumbar spine (15). Fortunately, the laparotomy approach identified that the tip of the screw was closed to the aorta, and surrounding structures were intact. However, once the vascular injury occurred, fatal consequences to the patient will be faced.

Iatrogenic IVC injury is a rare but life-threatening complication. To our knowledge, there was only three pedicle screw related IVC injuries reported in the literature. Wang et al. reported a case of IVC tear which occurred during posterior spinal fusion surgery (16). The patient underwent emergent exploratory laparotomy, and the torn vessel was repaired, but the patient died after two weeks later due to multiple-organ failure. Yen et al. presented a case with an L-3 pedicle screw pushed into the retroperitoneum and then migrated to IVC (17). A percutaneous endovascular technique was given to successfully retrieve the screw. In 2019, Makino et al. using endovascular treatment successfully managed a case of IVC and lumbar artery injury caused by tap insertion for a pedicle screw during lumbar interbody fusion (18). The latter two iatrogenic injuries have occurred in older patients, and lack of attention to poor bone quality may be the cause of this catastrophic event. In the present case, although bone mineral density examination was not performed, the patient is a 31-year-old young man, and osteoporosis may not be considered. During surgery, with a limited view of minimal invasive incision, the surgeon of a two-year trainee in the field of spinal surgery had difficulties putting the screwdriver bound to the screw end. After multiple attempts, he clockwise turned the screwdriver with forwarding pressure (in a wrong manner), leading to unexpectedly penetrating the anterior wall of the vertebral body and injuring the IVC.

Injury of the vessel was associated with a mortality rate high of 50% due to its catastrophic hemorrhage occurred (19). CTA or digital subtraction angiography (DSA), the excellent options to assess the intact of large blood vessels, have been mentioned as the basis of handle for this kind of issue in the literature (20, 21). After evaluation, it is particularly important to treat it individually. A management algorithm, open vascular surgery or endovascular surgery, has been introduced to make the best clinical decisions for PSF related vessel injuries (22, 23). Direct suture, segmental aortic reconstruction, and endovascular stent graft (ESG) implantation are available options for the treatment of the lesions (24–26). It is important to note that screw removal should be performed in a setting with the capacity for well gaining vascular control. However, maintaining the screw *in situ* without removal is also an alternative option in certain circumstances. lin et al. reported a case in which the vascular replacement surgery was performed without removing the screw for the stability of the spine (27). A similar case was reported by Saila et al. (28). In addition, Kenneth et al. recommended that surgeons needed to assess the risk of hardware removal for no symptoms patients since they found that no sequelae were caused by the screw that connected to the vessel at a long-term follow-up (29).

Although numerous cases of large vascular injury by implant have been listed, sparse literatures regarding the systematic management tactics for this extremely condition (5, 20, 22–24). After reviewing a large body of literature, the author summarized as follows: Follow-up periodically should be the main manner for implant of adjacent vessels. In addition, the implant involving vascular injury should be removed preventative under the circumstance of safety. Furthermore, the vital signs of patients should be fully monitored during the implantation or removal of internal fixation, and when there is unexplained hemorrhage or even blood pressure drop severely, it is necessary to consider anatomy-related major vascular injury. CTA or DSA as the primary tasks should be fully evaluated as early as possible for the formulation of the appropriate management approach.

## Conclusion

This present case suggested that although pedicle screw removal was a rather simple procedure, serious complications may arise from this procedure. Surgeons should keep vigilant to avoid the fatal complication noted in this case. Once it occurred during operation, an emergent diagnostic imaging scanning and an appropriate management approach are mandatory to make the best clinical decisions.

## Data Availability

The original contributions presented in the study are included in the article, further inquiries can be directed to the corresponding author.
